# High-Content, High-Throughput Analysis of Cell Cycle Perturbations Induced by the HSP90 Inhibitor XL888

**DOI:** 10.1371/journal.pone.0017692

**Published:** 2011-03-07

**Authors:** Susan K. Lyman, Suzanne C. Crawley, Ruoyu Gong, Joanne I. Adamkewicz, Garth McGrath, Jason Y. Chew, Jennifer Choi, Charles R. Holst, Leanne H. Goon, Scott A. Detmer, Jana Vaclavikova, Mary E. Gerritsen, Robert A. Blake

**Affiliations:** 1 Department of Molecular and Cellular Pharmacology, Exelixis, Inc., South San Francisco, California, United States of America; 2 Department of Genome Biology, Exelixis, Inc., South San Francisco, California, United States of America; University of South Florida College of Medicine, United States of America

## Abstract

**Background:**

Many proteins that are dysregulated or mutated in cancer cells rely on the molecular chaperone HSP90 for their proper folding and activity, which has led to considerable interest in HSP90 as a cancer drug target. The diverse array of HSP90 client proteins encompasses oncogenic drivers, cell cycle components, and a variety of regulatory factors, so inhibition of HSP90 perturbs multiple cellular processes, including mitogenic signaling and cell cycle control. Although many reports have investigated HSP90 inhibition in the context of the cell cycle, no large-scale studies have examined potential correlations between cell genotype and the cell cycle phenotypes of HSP90 inhibition.

**Methodology/Principal Findings:**

To address this question, we developed a novel high-content, high-throughput cell cycle assay and profiled the effects of two distinct small molecule HSP90 inhibitors (XL888 and 17-AAG [17-allylamino-17-demethoxygeldanamycin]) in a large, genetically diverse panel of cancer cell lines. The cell cycle phenotypes of both inhibitors were strikingly similar and fell into three classes: accumulation in M-phase, G2-phase, or G1-phase. Accumulation in M-phase was the most prominent phenotype and notably, was also correlated with TP53 mutant status. We additionally observed unexpected complexity in the response of the cell cycle-associated client PLK1 to HSP90 inhibition, and we suggest that inhibitor-induced PLK1 depletion may contribute to the striking metaphase arrest phenotype seen in many of the M-arrested cell lines.

**Conclusions/Significance:**

Our analysis of the cell cycle phenotypes induced by HSP90 inhibition in 25 cancer cell lines revealed that the phenotypic response was highly dependent on cellular genotype as well as on the concentration of HSP90 inhibitor and the time of treatment. M-phase arrest correlated with the presence of TP53 mutations, while G2 or G1 arrest was more commonly seen in cells bearing wt TP53. We draw upon previous literature to suggest an integrated model that accounts for these varying observations.

## Introduction

Cancer cells depend on an array of mutant and overexpressed proteins to support their unregulated growth and proliferation. However, this reliance on abnormal or highly expressed proteins strains the capacity of the cellular systems that support protein folding, and results in an increased dependence on molecular chaperones such as HSP90 [1], which is estimated to have more than 100 client protein substrates [2], [3]. Key cancer-related proteins such as AKT, ERBB2, and activated forms of EGFR and BRAF [4] are included in the HSP90 clientele, as are many other proteins with oncogenic associations. This preponderance of cancer-associated proteins in the HSP90 clients, combined with the overexpression of HSP90 in multiple tumor types [5], has led to a large number of preclinical and clinical studies focused on HSP90 inhibitors [6].

Because HSP90 is involved in a wide array of processes, its inhibition results in the simultaneous perturbation of multiple pathways and gives rise to complex cellular phenotypes. The most basic of these is a simple inhibition of proliferation, with varying degrees of subsequent cell death [4], [7], [8], [9]. However, the range of cell cycle effects induced by HSP90 inhibitors (accumulation in G1, G2, G2+M, or a combination of these, depending on the cell type) illustrates the diversity underlying the common phenotype of proliferation suppression [10], [11], [12], [13], [14], [15]. This phenotypic heterogeneity likely reflects genotype-specific responses to destabilization of the many cell cycle-associated HSP90 client proteins [16], including CDK1 and CDC25C [12], [13], CDK2/4/6 [17], [18], [19], WEE1 and CHK1 [20], [21], [22] and PLK1 [23]. Therefore, assessing the cell cycle phenotypes induced by small-molecule inhibitors of HSP90 can provide insight into the mechanisms by which loss of HSP90 function causes growth arrest and cell death, and can also potentially guide the selection of cancer types for the clinical application of HSP90 inhibitors.

Cell cycle analysis has traditionally been carried out by FACS (fluorescence-activated cell sorting) analysis of propidium iodide-stained cells, which assigns cell cycle phase by DNA content. However, FACS is limited by its inability to distinguish between G2 and M, by its imprecise quantification of S-phase, and in many cases, by its low throughput. To allow for more in-depth and easily scalable analysis of cell cycle phenotypes, we developed a novel and robust image-based cell cycle assay that accurately reports the phase status of a cell as well as its DNA content (2N vs. 4N). We surveyed a panel of 25 lung, breast, and melanoma cell lines and assessed the cell cycle perturbations induced by two distinct small-molecule inhibitors of HSP90: XL888, a novel synthetic small molecule and 17-AAG, an ansamycin derivative. Results showed that both HSP90 inhibitors induced remarkably similar cell cycle effects. We also observed phenotypic correlation with the mutational status of TP53, as well as unexpectedly complex behavior in the response of the cell cycle client PLK1 to HSP90 inhibition.

## Results

**Figure 1:**
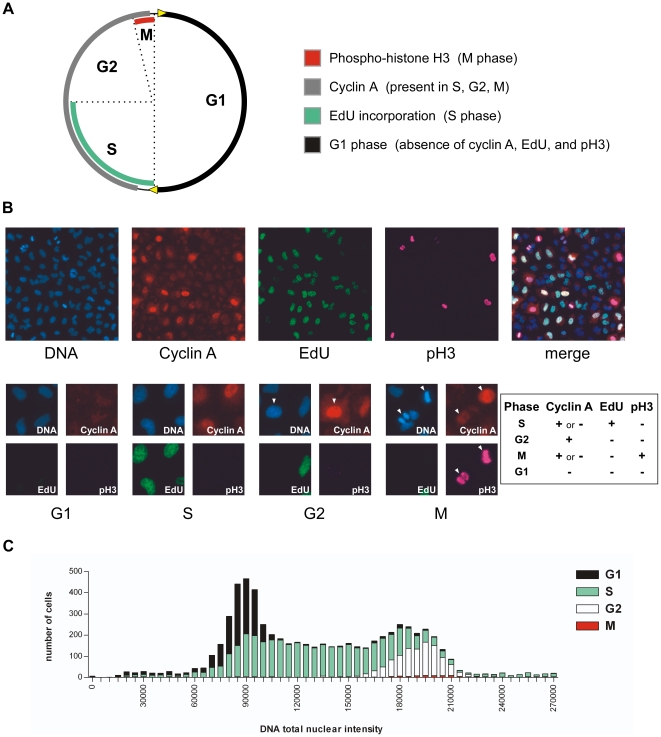
Development of the high-content (HC) cell cycle assay. (**A**) Markers used in the HC cell cycle assay and their distribution during the cell cycle. As defined by the HC cell cycle assay, G1 phase formally includes both G0 and G1 phases—however, for the sake of simplicity, we refer to it as “G1” rather than “G0/G1”. See text for discussion of assay development and validation. (**B**) Images of A549 cells stained for HC cell cycle analysis with Hoechst 33342, cyclin A, EdU, and pH3. Top panel, a field of asynchronous cycling cells; bottom panel, examples of G1, S, G2, and M cells. Bottom panels: the G2 panel shows one G2 cell (white arrowhead), one G1 cell, and one S cell; the M panel shows two M cells (white arrowheads) and three G1 cells. The G1 and S panels show exclusively G1 or S cells, respectively. The inset table summarizes the Boolean logic used to identify cell cycle phases when images are analyzed with the Cellomics Target Activation algorithm. (**C**) A DNA distribution plot of data derived from HC cell cycle analysis of DMSO-treated Calu-6 cells. This plot combines aspects of FACS (DNA content, as measured by total nuclear intensity of Hoechst 33342 staining) with the image-based cell cycle phase assignment, and demonstrates that the phase assignments correlate well with the DNA content expected for a given phase (i.e. G1 lies primarily at 2N; S lies between 2N and 4N, G2+M lies primarily at 4N). Note that complex karyotypes in some cell lines can contribute to a complex distribution, such that each phase is not completely contained within discrete boundaries of 2N-4N DNA content.

We developed a high-throughput, high-content, image-based cell cycle analysis method (Figure 1A–B) in which S-phase cells are defined by incorporation of the thymidine analog EdU (5-ethynyl-2′-deoxyuridine) into DNA, and M-phase cells are defined by immunostaining for the mitotic marker phospho-histone H3 (pH3) [24]. Immunostaining for cyclin A, which is present in S, G2, and M [25], allowed us to derive G1 and G2 phase assignments: G2 cells were defined as positive for cyclin A staining but negative for EdU and negative for pH3, while G1 cells were defined as negative for EdU, cyclin A, and pH3. To evaluate the accuracy of the phase designations, HeLa and A549 cells were synchronized with a double-thymidine block and released at timed intervals to create populations enriched for G1/S or G2/M. High-content cell cycle analysis showed the expected phase enrichments in these synchronized cells, as well as in asynchronous cells that were treated with taxol (paclitaxel) or hydroxyurea to enrich for M or for G1/S (data not shown). We also generated a “DNA distribution plot” histogram that combines a FACS-like display of DNA content with an overlay of cell cycle phase assignments (Figure 1C), and demonstrated that phase assignments in DMSO-treated Calu-6 cells were consistent with the expected 2N vs. 4N DNA content. DNA distribution patterns varied in different cell lines according to their degree of aneuploidy and heterogeneity, but the majority exhibited distinguishable 2N and 4N populations. (See Dataset S1 for an example of the custom Excel macro used to generate DNA distribution plots from raw cell cycle data output.)

**Figure 2:**
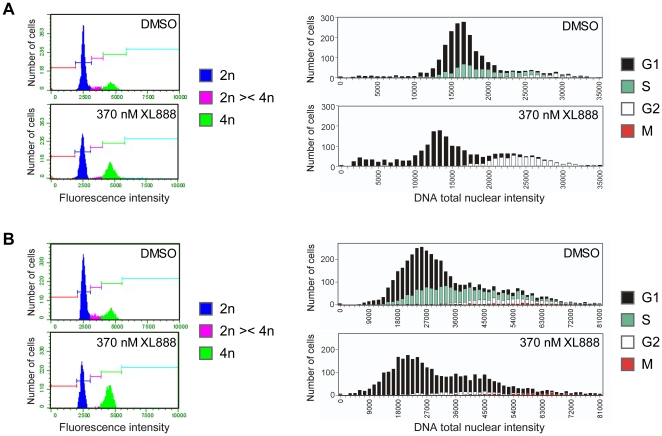
Comparison of HC cell cycle analysis to FACS analysis. (**A**) WM-266-4 cells were treated for 24 h with DMSO or 370 nM XL888: FACS analysis (left panel) of XL888-treated cells shows 4N accumulation and a decrease in S phase. HC cell cycle analysis (right panel) similarly shows that XL888 treatment resulted in G2 (4N) accumulation and loss of S cells, and also reveals the presence of a population of sub-2N dead/dying cells. (**B**) A375 cells were treated for 24 h with DMSO or 370 nM XL888. FACS analysis (left panel) of XL888-treated cells shows loss of S, some loss of 2N, and 4N accumulation. HC cell cycle analysis (right panel) similarly shows that XL888 treatment resulted in a loss of S and moderate loss of G1. However, HC analysis additionally revealed that the increase in 4N (seen in the parallel FACS analysis) is due to not to accumulation of G2 or M cells, but to the generation of 4N-pseudo-G1 cells, most likely by mitotic checkpoint slippage. Experiments were performed at least two times, and results from independent trials were consistent.

We used the high-content (HC) cell cycle method to analyze the cell cycle perturbations induced by XL888 (Figure S1A), a synthetic, orally bioavailable, ATP-competitive inhibitor of HSP90 with potent anti-proliferative activity against a large panel of cancer cell lines. We compared HC cell cycle analysis to FACS analysis in two XL888-treated melanoma lines: In WM-266-4 cells (Figure 2A), both methods showed that XL888 treatment caused loss of S-phase and accumulation of cells with 4N DNA content; the HC method additionally showed that the 4N accumulation consisted of G2 (and not M) cells. In XL888-treated A375 cells (Figure 2B), the HC cell cycle method revealed that the 4N accumulation seen by FACS analysis was not due to an increase in G2 or M, but to the generation of 4N-pseudo-G1 cells, presumably by mitotic checkpoint slippage (i.e. chromatin decondensation and mitotic exit without cytokinesis). These examples illustrate the greater clarity and higher definition of the HC method vs. traditional FACS analysis.

**Figure 3:**
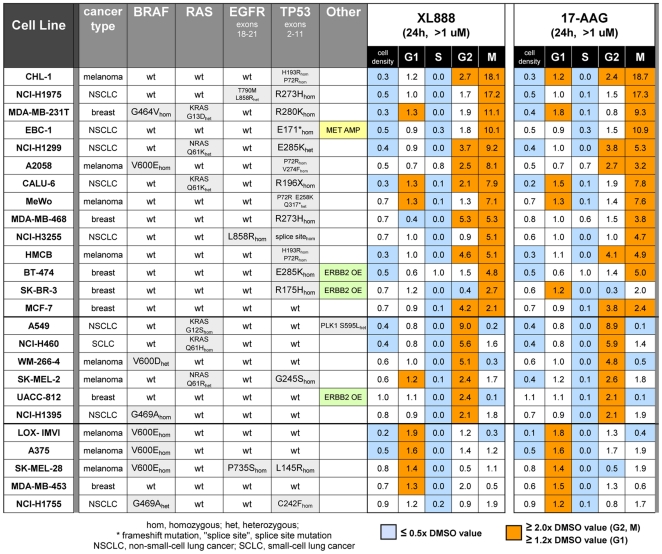
Cell cycle analysis of 25 cancer cell lines treated with XL888 or 17-AAG. Cells were treated with 1–1.6 uM XL888 or 17-AAG for 24 h, and cell cycle profiles were analyzed by the HC cell cycle method. Cell cycle data is normalized to the DMSO value for a given phase and given cell line and is represented as a fold-change vs. DMSO. The heat map color key is as follows: light blue, ≤0.5× DMSO value; orange, ≥2× DMSO value for G2 and M and ≥1.2× DMSO value for G1. Data is successively sorted in descending order of (1) accumulation in M, (2) accumulation in G2, and (3) accumulation in G1. Mutations are highlighted in gray. EBC-1 is a MET-amplified line (“MET AMP”) and BT-474, UACC-812, and SK-BR-3 are ERBB2-overexpessing lines (“ERBB2 OE”). Genotype data in this figure is derived from COSMIC [75] or from in-house sequencing. See Figure S1B for a version of the heat map that includes apoptosis and proliferation data as well as cell cycle profile data. Figure S1B also shows cell cycle phenotypes of cells treated with 0.4 uM XL888 or 17-AAG for 24 h, in comparison to the 1.1–1.67 treatment shown here. Chi-square analysis indicates that the probability of the observed correlation of mutant p53 lines with M+/−G2 status being random is 0.0089; see legend to Figure S1 for details.

We focused our analysis of the cell cycle effects of HSP90 inhibition on a panel of lung, breast, and melanoma cancer lines because of their dependence on key oncogenic drivers such as activated EGFR and BRAF, overexpressed ERBB2, and amplified MET—all of which are HSP90 client proteins [1]. We included 25 genetically diverse cancer cell lines in order to assess the effect of mutational status on the cell cycle response to XL888 or 17-AAG. Since the average proliferation IC_50_ across the cell panel was ∼0.1 uM (see Figure S1B), we chose ∼1 uM compound as the high end of the concentration curve in order to ensure a full range of response. Figure 3 summarizes the cell cycle phenotypes that resulted from 24 h of treatment with the highest tested concentration of each compound (1–1.6 uM), and illustrates the remarkably similar effects of both compounds. Note that the cell cycle profiles induced by treatment with a lower concentration of inhibitor (0.4 uM; see Figure S1B) closely resembled those seen at the 1 uM range, demonstrating that development of the cell cycle patterns shown in Figure 3 did not require high levels of XL888 or 17-AAG.

**Figure 4:**
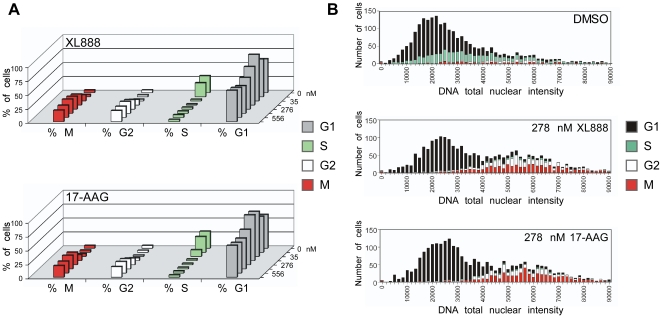
HC cell cycle analysis of HSP90 inhibitor-treated cells. MCF-7 cells were treated for 24 h with XL888 or 17-AAG at the indicated concentrations. HC cell cycle analysis is presented (**A**) as a bar chart showing %G1, S, G2, and M and (**B**) as a DNA distribution plot showing both DNA content and phase assignment. Note the “G1 blip” (see text) in the bar chart (A), and the loss of S and accumulation in M+G2 that is evident in both (A) and (B). Experiments were performed at least two times, and results from independent trials were consistent.

We observed three classes of cell cycle response to HSP90 inhibition: accumulation in M+/−G2 (M-class), accumulation in G2 (G2-class), and accumulation in G1 and/or 4N-pseudo-G1 (G1-class). The M-accumulation class was most common, and interestingly, 13 of the 14 M-class lines were mutant for TP53 (vs. only 3 TP53 mutants in the 11 non-M-class lines); we suggest a possible basis for this genotype-phenotype correlation in the Discussion. Figure 4 shows MCF-7 cells treated with XL888 or 17-AAG, and illustrates several characteristics of the M-class lines: a pronounced reduction in S with a concomitant increase in M (+/−G2), and a phenomenon we term the “G1 blip”—a distinctive biphasic pattern of G1 accumulation in which the percentage of G1 cells increased over a relatively narrow bracket at the lower end of the concentration range, then decreased at higher inhibitor concentrations (at which the percentage of M and G2 increased).

**Figure 5:**
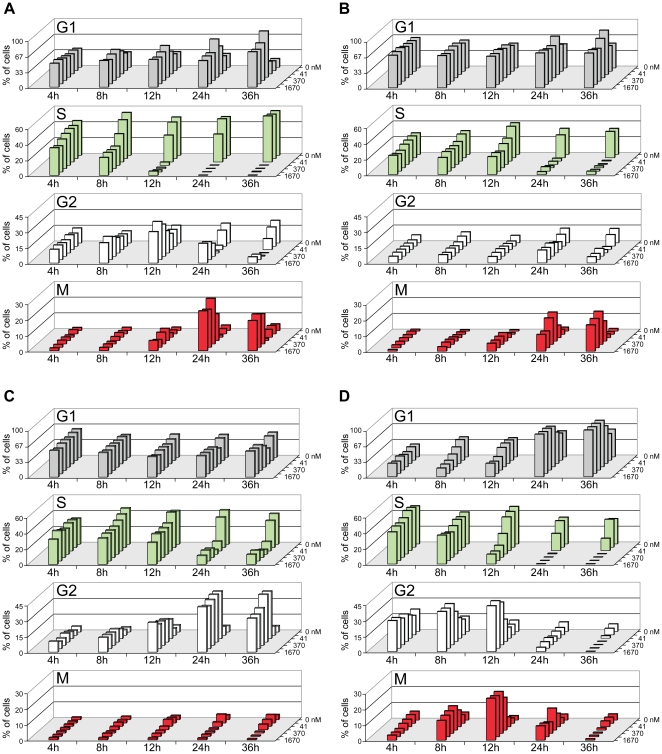
Timecourse analysis of cell cycle perturbations in XL888-treated cells. Cells were treated with the indicated concentrations of XL888 (14, 41, 123, 370, 1110, 1670 nM) at time = 0. Plates were then fixed at 4, 8, 12, 24, or 36 h and stained for HC cell cycle analysis. See text for discussion. (**A**) CHL-1 (**B**) EBC-1 (**C**) A549 (**D**) A375. For all three cell lines, 17-AAG effects were similar to those of XL888 (data not shown). Experiments were performed at least two times, and results from independent trials were consistent.

To examine the kinetics of these concentration-dependent changes in cell cycle perturbations, we carried out a timecourse analysis (Figure 5) in which representative cell lines from each phenotypic class were treated with XL888 and analyzed 4 h–36 h after compound addition. At 4–12 h, all tested lines showed varying degrees of proliferation inhibition, with maximal loss of S at 24–36 h. At 24–36 h, both of the M-class lines CHL-1 and EBC-1 showed the characteristic “G1 blip” at lower XL888 concentrations (Figure 5A–B), while at higher concentrations, there was prominent M accumulation as well as a decrease in G2 that was nearly the inverse pattern of the “G1 blip.” This complex concentration-dependent pattern may arise from loss of mitogenic signaling at lower concentrations, and inability to complete mitosis at higher concentrations. In A549 (G2-class) cells, accumulation in G2 was visible by 12 h (Figure 5C). However, at 24–36 h, higher concentrations of XL888 led to some loss of G2 with concomitant recovery of G1—suggesting that perhaps at higher concentrations, the integrity of the G2 (and M) checkpoints was compromised and some cells transited inappropriately through G2/M and into G1. The cell cycle profile of A375 cells (G1-class) was extremely dynamic (Figure 5D): At 4–12 h, G2 and M increased (with concomitant loss of G1 and S), but this increase was transient and was lost by 24 h. At 24–36 h, XL888 concentrations >40 nM resulted in an increase in G1 accompanied by a dramatic decrease in S. In these G1-class cells, the transition from G2+M accumulation at 12 h to G1 accumulation at 24 h indicates that a portion of the G2+M population was likely released from G2/M checkpoint surveillance and progressed to G1 and/or 4N-pseudo-G1 (via mitotic checkpoint slippage).

**Figure 6:**
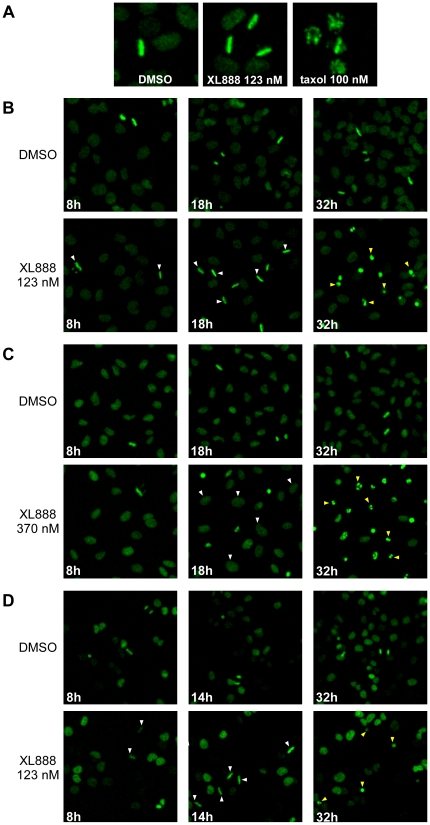
Timelapse analysis of XL888-treated cells. (**A**) Timelapse movie frames showing CHL-1 cells treated with DMSO, 123 nM XL888, or 100 nM taxol (paclitaxel) for 18 h. Note the different morphology of metaphase-arrested XL888-treated cells vs. prometaphase-arrested taxol-treated cells. (**B**) Timelapse movie frames showing CHL1 cells treated with DMSO or 123 nM XL888 for the indicated times. In this panel (B) as well as in panels (C) and (D), the same microscope field is shown at successive timepoints. In the 8 h and 18 h panels, some examples of XL888-treated cells displaying the linear, metaphase-like morphology are highlighted with white arrowheads; in the 32 h panel some examples of dead or dying cells are highlighted with yellow arrowheads. (**C**) Timelapse movie frames showing A549 cells treated with DMSO or 370 nM XL888 for the indicated times. In the 18 h panels showing XL888-treated cells, some examples of probable G2-arrested cells (based on cell size and lack of division) are highlighted with white arrowheads; in the 32 h panel, some examples of dead or dying cells are highlighted with yellow arrowheads (**D**) Timelapse movie frames showing A375 cells treated with DMSO or 123 nM XL888 for the indicated times. In the XL888-treated cells, some examples of the “linear quasi-metaphase” morphology are highlighted with white arrowheads (8 h, 14 h); some examples of dead or dying cells are highlighted with yellow arrowheads(32 h). For all three cell lines, 17-AAG effects were similar to those of XL888 (data not shown). Experiments were performed at least two times, and results from independent trials were consistent.

We used live-cell timelapse analysis to further characterize the cell cycle perturbations induced by HSP90 inhibition. CHL-1 (M-class), A549 (G2-class), and A375 (G1-class) cells were stably transfected with a histone-H2B-GFP plasmid to fluorescently mark chromatin, then treated with XL888 and imaged every 30 min for 36–48 h to track cell fate. Timelapse analysis (Figure 6A–B) revealed that XL888-treated CHL-1 cells arrested in M with highly organized chromosomes in a linear, metaphase-like configuration (as did other M-class cells; data not shown). It is notable that this metaphase-like phenotype was very different from the disorganized chromatin and prometaphase arrest that typically result from treatment with checkpoint-activating agents such as taxol (paclitaxel). The distinctive linear chromosome configuration in XL888-treated cells persisted for up to 16–18 h, although with increasing time, it became somewhat more disorganized, and some lagging chromosomes began to appear. Eventually, after prolonged M-arrest, CHL-1 cells underwent cell death without exiting from mitosis.

A549 cells (G2-class; Figure 6C) responded very differently to XL888 treatment: Most cells underwent one round of seemingly normal division, but then died 18–24 h after completion of the first division. The timing of cell death (and the increased size of the cells that subsequently died) suggested that after completion of the first division, cells were able to proceed through G1 and S and enter G2, but then were arrested and died in G2. XL888-treated A375 cells (G1-class; Figure 6D) also arrested in a metaphase-like configuration, although it was generally less organized than that of CHL-1, and the M arrest was more transient. A portion of the M-arrested A375 cells exited mitosis without cytokinesis to yield 4N-pseudo-G1 cells, and a portion died while in M-phase arrest. Some A375 cells did not accumulate in M; we judged these cells to bearrested in a 2N-G1 state, based on their lack of division and on their relatively small size.

**Figure 7:**
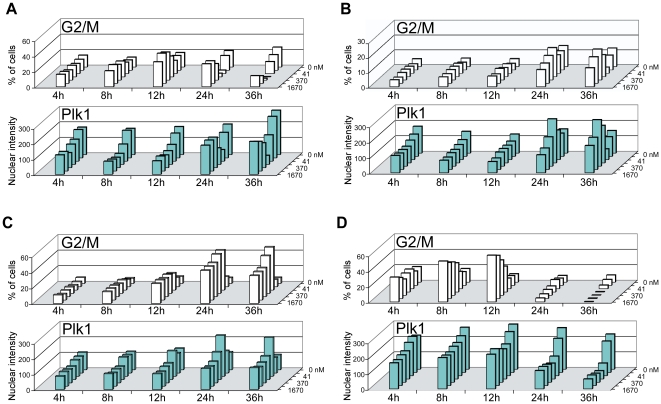
Timecourse analysis of cell cycle profiles and PLK1 levels. Cells were treated with the indicated concentrations of XL888 (14, 41, 123, 370, 1110, 1670 nM) at time = 0. Plates were then fixed at 4, 8, 12, 24, or 36 h and stained for a modified version of HC cell cycle analysis that allows for simultaneous detection of cell cycle phenotypes and of an additional marker protein, PLK1. For a given cell line, profiles of %G1 and %S were nearly identical to the %G1 and %S data shown in Figure 5, so those data are not displayed here; see Figure 5 for reference. See text for discussion. (**A**) CHL-1 (**B**) EBC-1 (**C**) A549 (**D**) A375. For all three cell lines, 17-AAG effects were similar to those of XL888 (data not shown). Experiments were performed at least two times, and results from independent trials were consistent.

We postulated that depletion of the client protein PLK1 could be contributing to the metaphase arrest phenotype. PLK1 is involved in entry into M, mitotic exit, and cytokinesis, and its depletion has been shown to result in an inability to complete mitosis [23], [26], [27]. To determine if reduced PLK1 levels correlated with M-phase accumulation, we used a modified version of the cell cycle assay (Figure 7) to simultaneously track PLK1 levels and cell cycle phase (G1, S, and G2/M) in a timecourse analysis of two M-class lines (CHL-1, EBC-1), one G2-class line (A549), and one G1-class line (A375). Because the G1 and S profiles in cells analyzed with this modified version of the assay were extremely similar to those in the standard assay (Figure 5), Figure 7 shows only PLK1 levels and the combined G2/M profile, rather than the complete G1-S-G2/M data set.

After 4–12 h of treatment with XL888, PLK1 levels in the M-class lines CHL-1 and EBC-1 (Figure 7A–B) decreased in a concentration-dependent manner, although the depletion was greater in CHL-1 (∼65% decrease) than in EBC-1 (∼40% decrease). At early timepoints, there was no particular correlation of PLK1 abundance with any phase, but after 24–36 h of treatment, PLK1 levels in both CHL-1 cells and EBC-1 cells tracked with the complex G2/M profile. Unexpectedly, after prolonged XL888-induced M and G2 accumulation, PLK1 levels *increased* relative to the same XL888 concentration at earlier timepoints—and in the case of EBC-1, surpassed the basal (DMSO) level. In A549 (G2-class) cells (Figure 7C), PLK1 was less sensitive to XL888—perhaps because of a mutation in the HSP90-binding C-terminal portion of PLK1 (see Figure 3), but PLK1 levels did correspond with the G2/M profile at later timepoints. In A375 (G1-class) cells (Figure 7D) there was clearly a concentration-dependent decrease in PLK1 levels at 4–8 h of treatment with XL888. Interestingly, at 12 h (the peak of G2+M accumulation) PLK1 levels no longer steadily decreased with increasing XL888 concentration, but instead reached a plateau at ∼100 nM XL888, with no further notable decrease at higher XL888 concentrations. However, at 24–36 h, when G2+M accumulation was lost and G1 accumulation predominated (see Figure 5D for G1 data), PLK1 levels again decreased substantially with increasing concentrations of XL888. We also detected PLK1 with a second antibody in all four cell lines; both antibodies yielded similar trends (data not shown).

**Figure 8:**
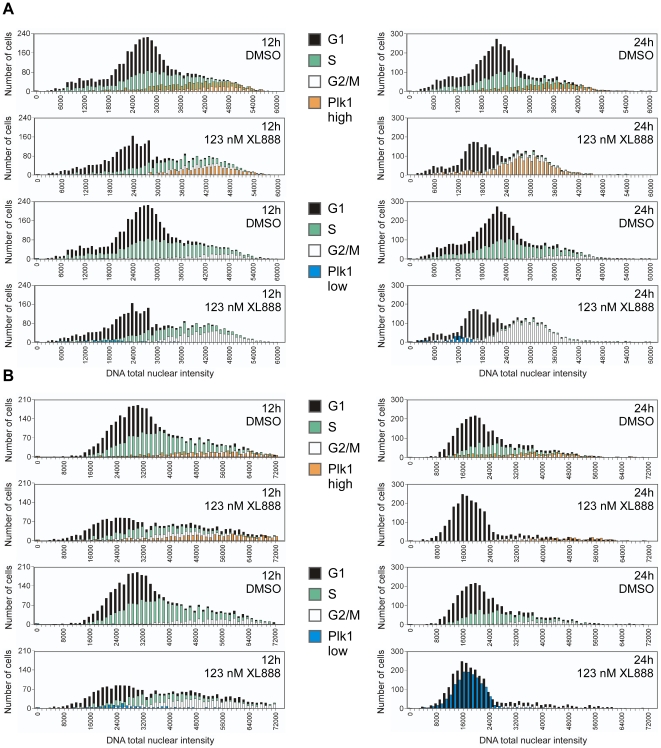
Co-analysis of cell cycle phases and PLK1 levels. (**A**) A549, (**B**) A375. Cells were treated with 123 nM XL888 for 12 h or 24 h. Plates were then fixed and simultaneously stained for PLK1 and for HC cell cycle analysis (in the same well). The plot shows the distribution of G1, S, and G2/M, represented as DNA content bins vs. number of G1, S, or G2/M cells per bin. This data is overlaid with bars that represent the distribution of high or low PLK1 levels, showing the ploidy and phase distribution of cells containing high or low levels of PLK1. The top panel in each section (A or B) shows the distribution of PLK1-high cells; the bottom panel shows the distribution of PLK1-low cells. The “high” cutoff represents cells that have PLK1 levels >1.5 times the median of the normal PLK1 distribution in DMSO-treated cells; the “low” cutoff is <0.5 times the median value. For both cell lines, 17-AAG effects were similar to those of XL888 (data not shown). Experiments were performed at least two times, and results from independent trials were consistent.

To investigate the relationship between PLK1 levels and cell cycle phase, we used the data from Figure 7 to create DNA distribution plots overlaid with PLK1 profiles that reflected the abundance of PLK1 in a given cell (“PLK1-high” and “PLK1-low”, see Figure 8). In the G2-class line A549, (Figure 8A) PLK1-high cells tracked with the 4N-G2/M population at 12 h and 24 h after XL888 addition, while the PLK1-low cells (although extremely scarce) tracked with 2N-G1. The G1-class line A375 (Figure 8B) similarly showed a correlation between PLK1-high cells and 4N-G2/M at 12 h (the peak of G2/M accumulation), with very few PLK1-low cells. However, by 24 h, when G1 was predominant, PLK1-low cells were abundant and tracked with the 2N-G1 population. Note that treatment of A375 cells with 123 nM XL888 (Figure 8B) yielded primarily 2N-G1 cells, while treatment with 370 nM XL888 (Figure 2B) yielded a mix of 2N-G1 and 4N-G1 cells; we will address this concentration-dependent phenotype development in the Discussion. In the M-class lines EBC-1 and CHL-1, phase correlations of PLK1-high (4N) and PLK1-low (2N) cells mirrored the general patterns observed in A549 and A375 (data not shown). These results are consistent with a model in which initial XL888-induced depletion of PLK1 contributes to accumulation in M+/−G2, and that if this state of 4N accumulation is prolonged, PLK1 is somehow desensitized to HSP90 inhibition.

## Discussion

The data presented in this study illustrate the complex effects of HSP90 inhibition on cell division: Cell cycle perturbation profiles were dependent on the concentration of HSP90 inhibitor, cell line genotype, and the duration of compound treatment. Previous studies have shown that inhibition of HSP90 causes a variety of cell cycle perturbations [10], [12], [15], [28], [29], and some reports have suggested correlations between genotype and the cell cycle phenotype induced by HSP90 inhibition [11], [30], [31]. However, such studies were often limited in scope with respect to the number of cell lines analyzed and to the extent of the tested concentration range of the HSP90 inhibitor. Therefore, we designed our analysis to include a genetically diverse panel of 25 cancer cell lines that were exposed to a wide concentration range of the HSP90 inhibitors XL888 and 17-AAG.

We found that the cell cycle perturbations induced by XL888 were remarkably similar to those of 17-AAG, indicating that the observed effects are the result of targeting HSP90 and are not due to scaffold-specific off-target activities. Cell cycle phenotypes of HSP90 inhibition fell into three classes: accumulation of cells in M+/−G2, in G2 alone, or in G1 (and/or 4N-pseudo-G1). M-accumulation was the most common phenotype, and in the M-class, cell cycle effects were highly concentration dependent: The distinctive “G1 blip” occurred at lower inhibitor concentrations (Figure 4, Figure 5), as did the IC_50_ for proliferation inhibition (Figure S1B), while accumulation in M+/−G2 (with attendant loss in G1) occurred at higher concentrations. The coincidence of the “G1 blip” and the proliferation IC_50_ at a similar concentration range suggests that HSP90 inhibitor-induced destabilization of growth-factor receptors disrupts downstream mitogenic signaling and prevents cells from proceeding through the G1 restriction point, leading to growth inhibition and G1 accumulation. This is supported by the sensitivity of growth-factor receptors such as MET, ERBB2, and mutant EGFR to relatively low concentrations of XL888 or 17-AAG ([32]; see also Figure S2). The degradation of HSP90 clients that function at the G1/S transition (such as CDK2, CDK4/6, and Cyclin D [17], [32], [33]) could also contribute to G1 accumulation.

The concentration-dependent shift from G1 accumulation to M+/−G2 accumulation in M-class lines likely reflects the incremental destabilization of increasing numbers of client proteins, with progressive loss of HSP90 function at increasing inhibitor concentrations. Many cell cycle-associated clients demonstrate differential sensitivity to HSP90 inhibition [16], [29], [34], [35], so as the level of HSP90 inhibition increases, the integrity of one checkpoint (G1) may be compromised, while other checkpoints (M, G2) are triggered. HSP90 function has previously been implicated in G2 and M by multiple studies showing that HSP90 is involved in the dynamics of centrosomes, kinetochores, and the mitotic spindle, and that inhibition of HSP90 causes abnormalities in these structures [34], [36], [37], [38], [39], [40], [41]. In addition, several proteins with roles in G2 or M are known to be HSP90 clients [16], [20], [21], [42] [43], [44], [45], [46]: CDK1 is essential for promoting entry into M-phase, CDC25 activates CDK1, PLK1 is involved in M and in the G2/M transition, survivin has roles in chromosome segregation and cytokinesis, and WEE1 and CHK1 police entry into M phase by inactivating the CDK1-cyclin B complex. Depletion of CDK1 or CDC25 would be predicted to result in G2 accumulation, and reduced levels of PLK1 [47] or survivin [48] generally result in M accumulation, while loss of WEE1 and CHK1 would be predicted to reduce the stringency of the G2/M checkpoint and promote inappropriate entry into M. M-phase accumulation was the predominant phenotype of HSP90 inhibition in our study, and it is noteworthy that almost all cell lines showing M+G2 accumulation were mutant for TP53, while the majority of the G2-only lines were wt for TP53—consistent with the requirement for p53 in activation of the G2 checkpoint after DNA damage or mitogen deprivation [49].

Interestingly, in the majority of the cells that underwent mitotic arrest in response to HSP90 inhibition, cells appeared to have completed metaphase, but were unable to successfully transit into anaphase. This implicated the HSP90 client PLK1 [23], [38], since its roles in mitotic exit and cytokinesis [27], [45], [50] are consistent with its destabilization resulting in the inability to complete mitosis. Depletion of PLK1 by RNAi leads to a variety of mitotic arrest phenotypes [26], [38], [51], [52], including some that mirror the metaphase-like chromosome morphology that we observed upon HSP90 inhibition (see data supplement to [26]).

Our observation that PLK1—although initially destabilized by XL888—could subsequently become desensitized to the continued presence of an HSP90 inhibitor was unexpected. Our analysis of PLK1 levels and cell cycle profiles in four cell lines showed a clear reduction in PLK1 levels in three wt PLK1 lines (CHL-1 and EBC-1, M-class; A375, G1-class) at early times after HSP90 inhibition. However, the A549 cell line (G2-class) was unusual in that PLK1 was only minimally destabilized by XL888. This lack of sensitivity is consistent with the finding by Simizu et al. [23] that C-terminal PLK1 mutations in A549 (and in some other cell lines; see [23]) interfere with HSP90 binding and result in lower basal levels of PLK1, presumably via loss of the stabilization that would normally result from the binding of HSP90 to PLK1. The A549 line used in our study had a heterozygous mutation in PLK1, S595L (Figure 3). Although not identical to the D457G PLK1 mutation found in A549 cells by Simizu et al., the S595L mutation does lie within the C-terminal HSP90-interaction domain [23], suggesting that it might similarly render PLK1 unable to interact strongly with HSP90, and lead to partial insensitivity of the mutant PLK1 to HSP90 inhibition.

In both the G2-class A549 line and the two M-class lines (CHL-1, EBC-1), the PLK1 abundance profile tracked with the G2+M profile after >12 h of treatment with HSP90 inhibitors (Figure 7). Prolonged persistence in M and/or G2 led to an apparent stabilization of PLK1, and high levels of PLK1 were found predominantly in cells with 4N DNA content (Figure 8), which is consistent with the known peak of PLK1 expression at the G2/M transition [50], [53]. Although the basis for the apparent desensitization of PLK1 to HSP90 inhibition is not clear, perhaps PLK1 is stabilized by protracted association with its mitotic binding partners [54] during prolonged G2/M arrest. Therefore, PLK1 would not be stabilized in cell lines that have a relatively short period of G2/M arrest (such as A375), consistent with our observations (see Figure 5D, Figure 7D).

The phenotype of HSP90 inhibition in A375 cells was intriguing because of the evolution of the XL888-induced G2+M accumulation into G1 accumulation over time (Figure 5D). A375 (see Figure 2B) and LOX-IMVI (data not shown) were the only two lines in our analysis in which HSP90 inhibition led to the generation of a population of 4N-pseudo-G1 cells, presumably via mitotic checkpoint slippage. The mutational status of these two lines is particularly relevant to interpretation of the data, since BRAF in both A375 and LOX-IMVI is constitutively activated by the V600E mutation [55]. Recent studies have linked BRAF to mitotic checkpoint regulation [56], [57], and BRAF also promotes localization of the checkpoint proteins BUB1, MAD2, and TTK (MPS1) to unattached kinetochores [58]. Interestingly, TTK, which is essential for checkpoint signaling [59], [60], is also directly affected by the mutational status of BRAF: TTK is destabilized when BRAF is depleted, but is stabilized by BRAF V600E, resulting in checkpoint hyperactivation [61].

It is important to note that although wt BRAF is relatively insensitive to HSP90 inhibition, BRAF V600E begins to be destabilized after 8–12 h of treatment with 1 uM 17-AAG, with nearly complete loss at 24 h [62], [63] (see also Figures S3, S4). Taken together, this suggests that the shift from G2+M to 4N-pseudo-G1 accumulation in BRAF V600E lines might be explained as follows (for supporting data, see Figure S4, a timecourse immunoblot analysis of A375 and LOX-IMVI cells treated with 370 nM XL888): At early timepoints (e.g. 4–12 h) after treatment with HSP90 inhibitors, degradation of cell cycle-associated clients such as CDK1 and PLK1 causes accumulation in G2 (CDK1) and M (PLK1); whether a cell is trapped in G2 or M would depend on its p53 status and on its position in the cell cycle at the time at which CDK1 or PLK1 levels became limiting. WEE1 and CHK1, which regulate the G2 exit checkpoint by inactivating the CDK1-cyclin B complex [43], [44] are maximally destabilized at ∼12–24 h in the V600E lines A375 and LOX-IMVI, and this loss likely allows cells to exit G2 and inappropriately enter M. This is consistent with our timecourse data showing loss of G2 accumulation and an increase in M accumulation between 12–24 h (Figure 5D, 7D). At this 12 h–24 h timeframe, these cells are most likely not able to successfully exit M because of the destabilization of clients such as PLK1 [23], [38], which has roles in mitotic exit and cytokinesis [27], [50] (see Figure 7D for a timecourse analysis of PLK1 levels in XL888-treated A375 cells).

However, at 24–36 h of XL888 treatment, A375 cells show a loss of M accumulation and an increase in G1/4N-G1, which may explained as follows: BRAF V600E is also maximally degraded after 12–24 h of treatment with HSP90 inhibitors [62], [63]; also see Figure S4). Keeping in mind that the mitotic checkpoint kinase TTK is destabilized when BRAF is depleted [61], and that loss of TTK function impairs checkpoint control and leads to premature mitotic exit [64], we suggest that at these later timepoints, the loss of BRAF V600E and the consequent destabilization of TTK contribute to mitotic checkpoint slippage of the pool of M-arrested cells, generating 4N-pseudo-G1 cells. This model provides an explanation for the shifting patterns of the A375 cell cycle profile from G2+M at 4–12 h of XL888 treatment to 4N-G1 at 24–36 h (see Figure 5D; see also Figure 2B for a DNA-distribution plot of A375 cells treated with 370 nM XL888 for 24 h). The overall scenario we propose is also consistent with our observation that of the 25 lines analyzed in this study, a 4N-pseudo-G1 population was found only in the BRAF V600E lines A375 and LOX-IMVI (Figure 2 and data not shown).

The most prominent genotype/phenotype correlation that we observed in this study was that of TP53 mutant status with the M accumulation induced by HSP90 inhibition. The primary role of p53 is to prevent the growth of cells that are not fit to replicate, and it coordinates a response to DNA damage or mitogen deprivation by imposing cell cycle blockades to prevent division [49], [65]. p53 functions in part through transactivation of its transcriptional target p21, which inhibits cyclin/CDK complexes and thereby controls the G1/S and G2/M transitions [66]. However, in cancer cells, p53 activity is frequently compromised, and the majority of the known p53 mutations cause loss of function and impair its transcriptional activity to varying extents [67]. One consequence of the loss of p53 function is an ancillary loss of p21 function: Most loss-of-function p53 mutant cells are expected to have intrinsically low levels of p21, as has been shown experimentally for some tumors and cell lines (e.g. [68], [69], [70]; also data not shown). So in these TP53 mutant lines, the loss of p53 function and subsequent reduction or loss of p21 activity would undermine proper regulation of the G1/S and G2/M phase transitions, resulting in weakened checkpoints even in an unperturbed cell.

In this context, it is particularly notable that HSP90 is known to be important in maintaining the functionality of mutated p53: Several studies have shown that mutant p53 has greater reliance on HSP90 than wt p53 and that the mutant form is destabilized by HSP90 inhibition [31], [71], [72], [73]. Sugimoto et al. [22] and Tse et al. [35] have also shown that in p53 mutant cells (but not p53 wt cells), HSP90 inhibition abrogated a DNA-damage-induced G2 checkpoint arrest and allowed cells to transit into M. This loss of robust checkpoint control and subsequent escape from G2 arrest is likely due at least in part to destabilization of mutant p53, combined with a cellular state in which there is insufficient p21 activity (both intrinsically and via the HSP90 inhibitor-mediated loss of mutant p53) to enforce the checkpoints.

The correlation between M-phase accumulation and TP53 mutant status that we observed is consistent with this overall model: Destabilization of mutant p53 combined with the corresponding loss of p21 activity and the subsequent weakening of G1/S and G2/M checkpoint surveillance could permit cells to inappropriately enter M, as would destabilization of WEE1 and CHK1. However, although TP53 mutant status would allow these cells to elude the G1 and G2 checkpoints, they would be unable to complete mitosis because of the degradation of PLK1 and/or other cell cycle-associated clients. In contrast, we found that HSP90 inhibition in wt TP53 lines generally resulted in G2 or G1 accumulation. This is consistent with a scenario suggested by Lin et al. [31] that incorporates the following observations: MDM2, a negative regulator of p53, is normally activated and stabilized by AKT-mediated phosphorylation [74]. HSP90 inhibitor-induced destabilization of the HSP90 client AKT [1] results in decreased levels of MDM2, and subsequently, results in de-repression of wt p53 and increased expression of the p53 target p21. p21 then inhibits CDK/cyclin complexes, restricting G1-to-S and G2-to-M transit and leading to accumulation in G1 and G2 [44]. The AKT-p53-p21 axis is, of course, not the only means by which the cell cycle is disrupted upon treatment with an HSP90 inhibitor—the inhibitor-induced disruption of normal cell cycle transit is also due to the destabilization of proteins such as EGFR, MET, CDK1, PLK1, and others.

In summary, we propose the following overall model to integrate our current observations with previous studies: In most cell lines, relatively low levels of HSP90 inhibitors induce G1 accumulation through destabilization of sensitive growth-factor receptors and subsequent loss of mitogenic signaling. At higher concentrations of HSP90 inhibitors, TP53 status is an important determinant of cell fate: HSP90 inhibition destabilizes mutant p53, compromising the G1 and G2 checkpoints and allowing cells to transit into M, although the ability to successfully complete M-phase is compromised by degradation of cell cycle clients such as PLK1, leading to M-phase accumulation. Conversely, in TP53 wt cells, the G1 and G2 checkpoints remain relatively robust, preventing inappropriate cell cycle progression and resulting in G1 and G2 accumulation.

## Materials and Methods

### Materials

Cell lines were obtained from ATCC (Manassas, VA) and maintained in ATCC-specified media containing 10% FBS. 17-AAG and taxol (paclitaxel) were obtained from EMD Chemicals (Gibbstown, NJ) and XL888 from Exelixis, Inc. (South San Francisco, CA).

### High-content cell cycle analysis

Cells were seeded 14–18 h prior to compound addition, and cells were ∼40–50% confluent and in log phase at the time of treatment. Compounds were serially diluted in DMSO, then diluted to 5× in serum-free medium and added to cells to yield a 1× final concentration. Final DMSO concentrations did not exceed 0.5%. 20 uM EdU (A10044; Invitrogen, Carlsbad, CA) was added for 30 min prior to fixation with 3.7% formaldehyde. Cells were washed in PBS, permeabilized for 15 min with 0.5% Triton X-100 in PBS, washed in PBS, and blocked for 10 min with 3% BSA in PBS. To label incorporated EdU, cells were incubated for 30 min with TBS, pH 7.2 containing 4 mM CuSO4, 1.94 mg/ml sodium ascorbate, and 4.5 ug/mL Alexa-fluor 488-azide (Invitrogen A10266), washed with PBS, and incubated overnight at 4°C with primary antibodies in 1% BSA/PBS (Cyclin A, ab16726, Abcam, Cambridge, MA; phospho-histone H3, #3377, Cell Signaling Technology, Danvers, MA). Cells were then washed with PBS and incubated 2–4 h with Alexa-fluor-labeled secondary antibodies (Invitrogen A11010, A21244) and Hoechst 33342 (Invitrogen H3570). Cells were then imaged with a Cellomics Arrayscan VTI (Thermo, Pittsburgh, PA) (XF93 optical filter set) at 10× magnification using the Target Activation Bioapplication (with Boolean events designated by the Event Wizard module). To generate DNA distribution plots, DNA content data and cell cycle phase assignments were analyzed using a custom Excel macro (see Text S1).

### Combined PLK1 immunofluorescence/cell cycle analysis

An alternate method of cell cycle analysis omitted the phospho-histone H3 antibody, allowing an additional marker protein of interest (PLK1, Abcam ab47867) to be examined simultaneously with the cell cycle staining. Cell cycle phases were defined as follows: S-phase, positive for EdU incorporation; combined G2/M-phase, positive for Cyclin A but negative for EdU; G1 phase, negative for EdU and negative for cyclin A. PLK1 was also detected with a second, independent antibody (Abcam ab17056) to ensure that the observed results were not antibody-specific.

### Fluorescence-activated cell sorting (FACS) analysis

Cells were treated with compound for 24 h or 48 h in complete medium, then harvested, fixed in ethanol, and analyzed on the Guava EasyCyte flow cytometer (Millipore, Billerica, MA) using propidium iodide staining.

### Live-cell timelapse analysis

Stable histone-H2B-GFP lines were generated by blasticidin selection of cells transfected with the pBOS-H2B-GFP plasmid (559241, BD Biosciences, San Jose, CA). Cells were imaged in a 96-well plate using the live-cell chamber of a Cellomics Arrayscan VTI (XF100 optical filter set) at 10× magnification.

## Supporting Information

Figure S1 **Cell cycle analysis and proliferation/apoptosis IC_50_/EC_50_ analysis of 25 cancer cell lines treated with XL888 or 17-AAG.** (**a**) Structure of XL888 (**b**) Cell lines were treated for 24 h with either 0.4 uM or 1–1.6 uM XL888 or 17-AAG and stained for HC cell cycle analysis; doubling time was determined by cell counting. In a separate experiment, proliferation IC**_50_** and apoptosis EC_50_ values were determined as noted in Supplemental Materials and Methods. Cell cycle data is normalized to the DMSO value for a given phase and given cell line and is represented as a fold-change vs. DMSO. Heat map color key is as follows: light blue, ≤0.5× DMSO value; orange, ≥2× DMSO value for G2 and M and ≥1.2× DMSO value for G1. Data is successively sorted in descending order of (1) accumulation in M, (2) accumulation in G2, and (3) accumulation in G1. Mutations are highlighted in gray. EBC-1 is a MET-amplified line (“MET AMP”) and BT-474, UACC-812, and SK-BR-3 are ERBB2-overexpessing lines (“ERBB2 OE”). Genotype data in this table is derived from COSMIC [75] or from in-house sequencing. A chi-square analysis of the apparent correlation of p53 mutant status with the M+/−G2 phenotype revealed that the probability that the observed distribution is the same as the random distribution is 0.0089: In this case of 20 cell lines characterized as having either an M+/−G2 phenotype (n = 14) or a G2-only phenotype (n = 6), if the mutant p53 cell lines (14 of 20; 70%) were distributed randomly between the two groups, 9.8 of the 14 M+/−G2 lines would have mutant p53 (vs. 13 of 14 observed to have mutant p53), and 4.2 of the G2-only lines would have mutant p53 (vs. 1 of 6 observed to have mutant p53). The chi-square value for the difference between the expected vs. observed distribution is 11.61, with three degrees of freedom.
(TIF)

Figure S2 **Client protein analysis: XL888-treated lung and breast cancer cells.** Cells were treated for 24 h with XL888 at the indicated concentrations. Cell lysates were then immunoblotted for EGFR, MET, and ERBB2. (**a**) A549 (EGFR wt), EBC-1 (MET amplified), and NCI-H1975 (EGFR T790M/L858R). (**b**) MCF-7, SK-BR-3 (ERBB2-overexpressed).
(TIF)

Figure S3 **Client protein analysis: XL888-treated melanoma cells.** (**a**) A375 and SK-MEL-2 cells were treated for 24 h with XL888 at the indicated concentrations. Cell lysates were then immunoblotted for CRAF, BRAF, p-ERK, and total ERK. (**b**) The inset table shows calculated IC_50_ values for XL888-induced degradation (BRAF, CRAF) or inhibition of phosphorylation (p-ERK).
(TIF)

Figure S4 **Timecourse of client protein analysis: XL888-treated melanoma cells.** The BRAF V600E mutant cell lines (**a**) A375 and (**b**) LOX-IMVI were treated with 370 nM XL888, and cells were harvested at the indicated timepoints. Cell lysates were then immunoblotted for BRAF, CDK1, and CHK1, and the resultant blots were quantified to assess XL888-induced protein degradation, as shown in the corresponding graph.
(TIF)

Text S1 **Supplemental Materials and Methods**
(DOC)

Dataset S1 (XLS)
