# Correction: High-Content, High-Throughput Analysis of Cell Cycle Perturbations Induced by the HSP90 Inhibitor XL888

**DOI:** 10.1371/annotation/73d83e95-8f14-48ed-bb67-2310a33e4ecc

**Published:** 2011-03-21

**Authors:** Susan K. Lyman, Suzanne C. Crawley, Ruoyu Gong, Joanne I. Adamkewicz, Garth McGrath, Jason Y. Chew, Jennifer Choi, Charles R. Holst, Leanne H. Goon, Scott A. Detmer, Jana Vaclavikova, Mary E. Gerritsen, Robert A. Blake

The 7th and 11th authors were incorrectly assigned two affiliations. Jennifer Choi and Jana Vaclavikova are solely affiliated with: "Department of Genome Biology, Exelixis, Inc., South San Francisco, California, United States of America."

