# Supplementary figures and images for: High-Content, High-Throughput Analysis of Cell Cycle Perturbations Induced by the HSP90 Inhibitor XL888
Source: PLoS One. 2011 Mar 7;6(3):e17692. doi: 10.1371/journal.pone.0017692 (PMC3049797; doi:10.1371/journal.pone.0017692)

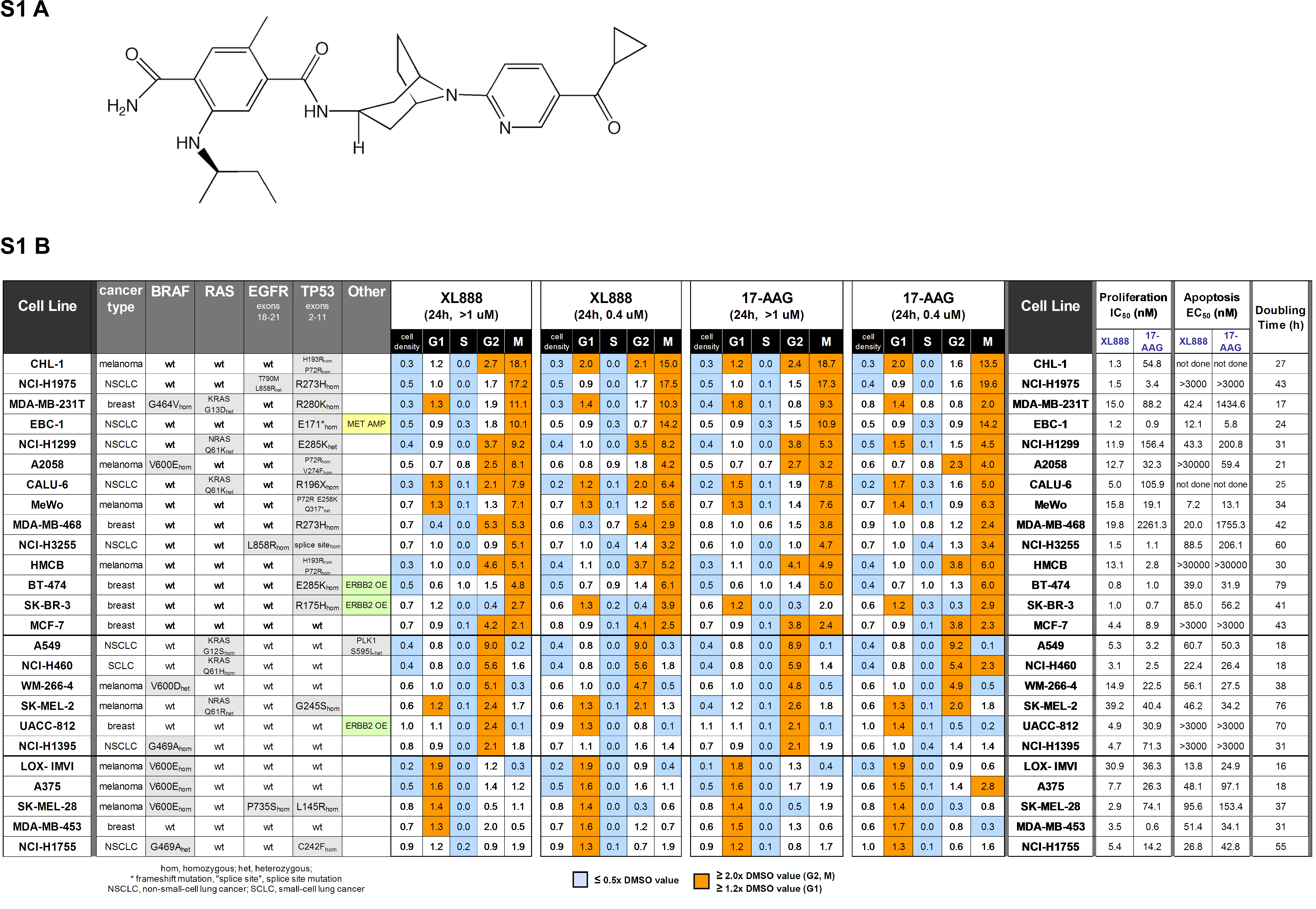

Supplement: Figure S1 — Cell cycle analysis and proliferation/apoptosis IC50/EC50 analysis of 25 cancer cell lines treated with XL888 or 17-AAG. (a) Structure of XL888 (b) Cell lines were treated for 24 h with either 0.4 uM or 1–1.6 uM XL888 or 17-AAG and stained for HC cell cycle analysis; doubling time was determined by cell counting. In a separate experiment, proliferation IC50 and apoptosis EC50 values were determined as noted in Supplemental Materials and Methods. Cell cycle data is normalized to the DMSO value for a given phase and given cell line and is represented as a fold-change vs. DMSO. Heat map color key is as follows: light blue, ≤0.5× DMSO value; orange, ≥2× DMSO value for G2 and M and ≥1.2× DMSO value for G1. Data is successively sorted in descending order of (1) accumulation in M, (2) accumulation in G2, and (3) accumulation in G1. Mutations are highlighted in gray. EBC-1 is a MET-amplified line (“MET AMP”) and BT-474, UACC-812, and SK-BR-3 are ERBB2-overexpessing lines (“ERBB2 OE”). Genotype data in this table is derived from COSMIC [75] or from in-house sequencing. A chi-square analysis of the apparent correlation of p53 mutant status with the M+/−G2 phenotype revealed that the probability that the observed distribution is the same as the random distribution is 0.0089: In this case of 20 cell lines characterized as having either an M+/−G2 phenotype (n = 14) or a G2-only phenotype (n = 6), if the mutant p53 cell lines (14 of 20; 70%) were distributed randomly between the two groups, 9.8 of the 14 M+/−G2 lines would have mutant p53 (vs. 13 of 14 observed to have mutant p53), and 4.2 of the G2-only lines would have mutant p53 (vs. 1 of 6 observed to have mutant p53). The chi-square value for the difference between the expected vs. observed distribution is 11.61, with three degrees of freedom. (TIF) [file pone.0017692.s001.tif]

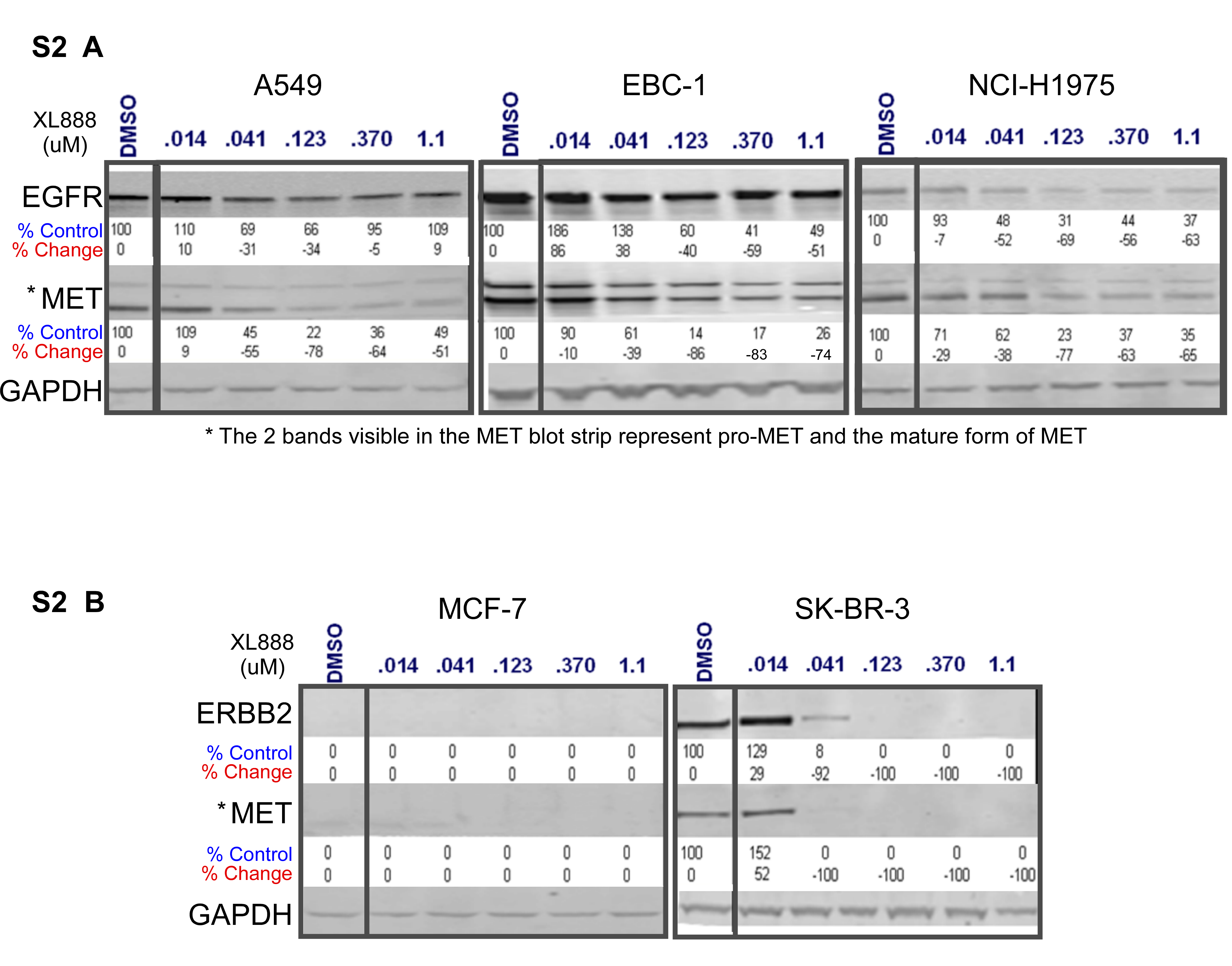

Supplement: Figure S2 — Client protein analysis: XL888-treated lung and breast cancer cells. Cells were treated for 24 h with XL888 at the indicated concentrations. Cell lysates were then immunoblotted for EGFR, MET, and ERBB2. (a) A549 (EGFR wt), EBC-1 (MET amplified), and NCI-H1975 (EGFR T790M/L858R). (b) MCF-7, SK-BR-3 (ERBB2-overexpressed). (TIF) [file pone.0017692.s002.tif]

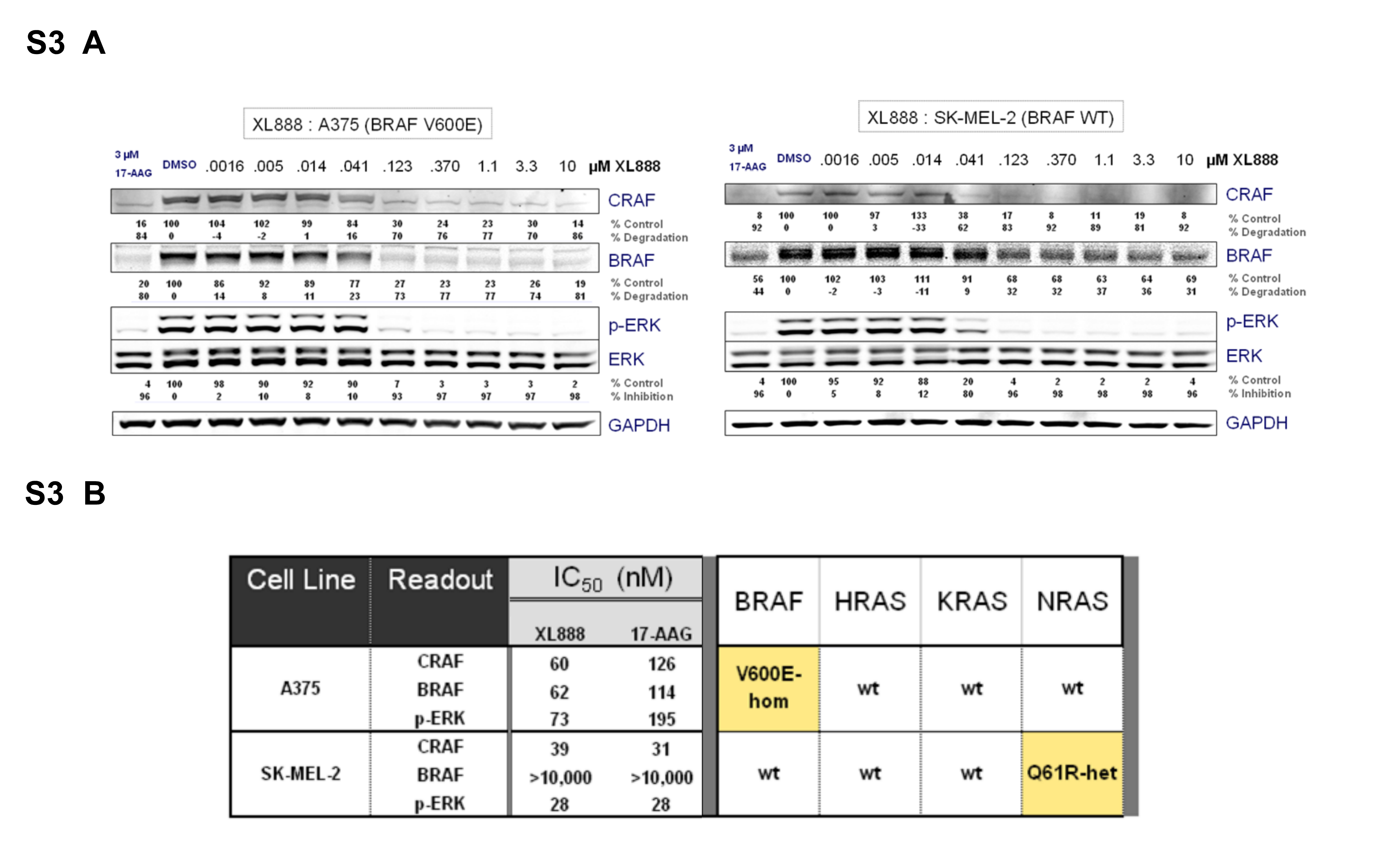

Supplement: Figure S3 — Client protein analysis: XL888-treated melanoma cells. (a) A375 and SK-MEL-2 cells were treated for 24 h with XL888 at the indicated concentrations. Cell lysates were then immunoblotted for CRAF, BRAF, p-ERK, and total ERK. (b) The inset table shows calculated IC50 values for XL888-induced degradation (BRAF, CRAF) or inhibition of phosphorylation (p-ERK). (TIF) [file pone.0017692.s003.tif]

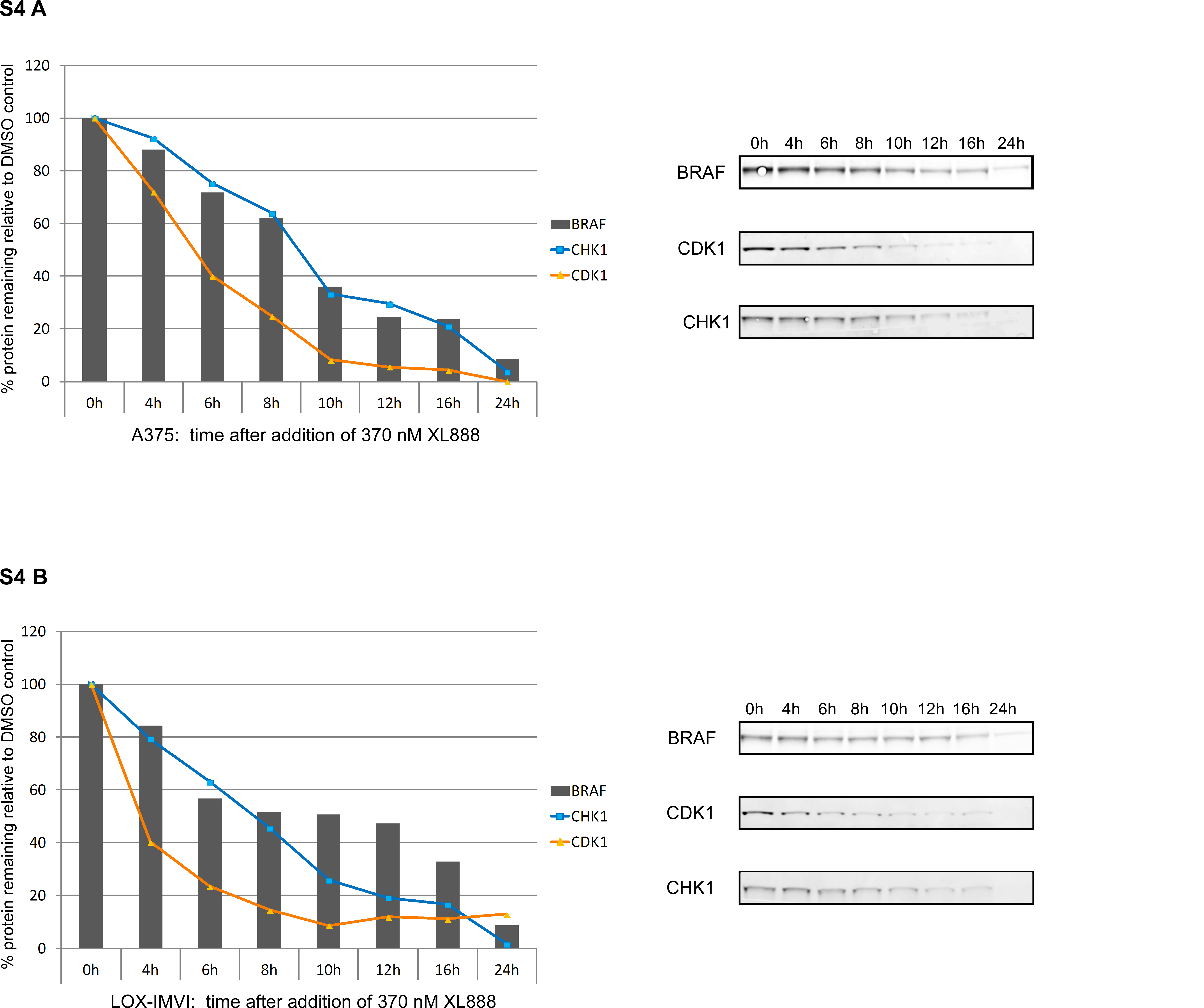

Supplement: Figure S4 — Timecourse of client protein analysis: XL888-treated melanoma cells. The BRAF V600E mutant cell lines (a) A375 and (b) LOX-IMVI were treated with 370 nM XL888, and cells were harvested at the indicated timepoints. Cell lysates were then immunoblotted for BRAF, CDK1, and CHK1, and the resultant blots were quantified to assess XL888-induced protein degradation, as shown in the corresponding graph. (TIF) [file pone.0017692.s004.tif]
