# Supplementary material for: High-Content, High-Throughput Analysis of Cell Cycle Perturbations Induced by the HSP90 Inhibitor XL888
Source: PLoS One. 2011 Mar 7;6(3):e17692. doi: 10.1371/journal.pone.0017692 (PMC3049797; doi:10.1371/journal.pone.0017692)
Supplement: Text S1 — Supplemental Materials and Methods (DOC) [file pone.0017692.s005.doc]

**Supporting Information: Cell Cycle Effects of HSP90 Inhibition; Lyman et al.**

**Supplemental Materials and Methods**

**DNA sequencing**: Genomic DNA was isolated using the DNeasy kit (69581, Qiagen, Valencia, CA). The regions of interest (EGFR exons 18-21, PLK1 exons 8-9 and partial exon 10, and full length TP53) were amplified using TaKaRa LA Taq DNA Polymerase (RR002A, TaKaRa Bio / Chemicon International Inc., Temecula, CA) and the resulting PCR products were sequenced using the same primers. Sequence analysis was done with Mutation Surveyor (Softgenetics, State College, PA).  Primer sequences are listed below:

| **Gene** | **Exon** | **Forward primer sequence** | **Reverse Primer Sequence** |
| --- | --- | --- | --- |
| EGFR | 18 | TTCCAAATGAGCTGGCAAGT | TGGAGTTTCCCAAACACTCAG |
| EGFR | 19 | CCCCAGCAATATCAGCCTTA | GCCAGTGCTGTCTCTAAGGG |
| EGFR | 20 | CTCTCCCACTGCATCTGTCA | GATGGGACAGGCACTGATTT |
| EGFR | 21 | ACATCTTCTTTCATGCGCCT | AAGCAGCTCTGGCTCACACT |
| PLK1 | 8,9 | GTGCTGGAATCACAGGCATG | CTCCACATTCAACCCACATG |
| PLK1 | 10 | TGTGGGTTGAATGTGGAGTG | GTGCCATGTCTGCAGTGTGC |
| TP53 | 2,3 | CTCAGACACTGGCATGGT | GGACTGTAGATGGGTGAAAA |
| TP53 | 4 | AAACAACGTTCTGGTAAGGA | GAAGAGGAATCCCAAAGTTC |
| TP53 | 5,6 | GTTCACTTGTGCCCTGACTTTC | CACCTGGAGGGCCACTGACAA |
| TP53 | 7 | TTGCCACAGGTCTCCCCAA | AAGAAATCGGTAAGAGGTGG |
| TP53 | 8,9 | GACAAGGGTGGTTGGGAGT | GAGGTCCCAAGACTTAGTACCTG |
| TP53 | 10 | GTTGCTTTTGTACCGTCATA | AGAATGGAATCCTATGGCTT |
| TP53 | 11 | GTAAACATATTTGCATGGGG | GGACAAAGCAAATGGAAGT |

**Excel macro analysis for DNA distribution plots**: HC cell cycle distribution plots were generated using an Excel based visual basic macro. Individual cell data were exported from Cellomics vHCS software to Excel with the following data types and export order: Event Type Profile (numeric descriptor indicating which cell cycle phase a cell was assigned to: 1=G1, 0=S, 2=G2, 3=M), EventType1Status, EventType2Status, EventType3Status (binary data with 1 indicating that the cell is positive for each status and 0 indicating that the cell is negative for each status), ObjectTotalIntenCh1 (the summation of pixel intensity values for Hoechst 33342 staining in the nuclear region of each cell).

The visual basic macro was designed to generate distribution plots by grouping cellular data from a single 384 well plate according to the conditions of treatment, then to bin each cell within a treatment according to its Hoechst 33342 staining intensity (ObjectTotalIntenCh1), and finally to determine the number of cells in each bin that fall into each cell cycle class using the EventTypeProfile data. The visual basic script stepped sequentially through the list of data for a given treatment type (listed on the “InputDataSheet” tab of the Excel workbook), using a separate Excel workbook tab (“Distribution”) to record a running total of the number of cells counted for each Hoechst 33342 staining intensity bin. Counts were accumulated within each bin, giving rise to the running total of cells in each cell cycle phase designation (EventType). A different Excel row was used for each intensity bin and, within that row, a different column was used to count up the number of cells in each cell cycle phase designation for that intensity bin.

Graphs were generated using a “stacked column” format with the x-axis representing each Hoechst 33342 staining intensity bin and each section of the stacked column representing the number of cells in each cell cycle phase designation. Estimates of the boundaries between <2N, 2N, 4N and >4N were calculated based on (1) the position of the maximal peak of the distribution of DMSO-treated cells (assumed to be 2N peak), (2) the inflection point following the 2N peak, the assumption that the 4N peak is twice the 2N peak intensity, (3) the region of the distribution where S phase cells predominate over G1 phase cells, and (4) the peak of combined G2 and M phase cells. Manual curation of these boundaries was occasionally required depending on the shape of the distribution and how much it deviated from an ideal distribution (depending on the degree of aneuploidy and heterogeneity for a given cell line). Percentages of <2N, 2N, 4N and >4N were calculated from these boundaries.

A copy of the Excel workbook with embedded visual basic macro and example data is available for download in the online supplement (**Dataset S1**; Excel cell cycle analysis macro example). In order for the macro to function, the following custom sort list must be added to your local copy of Excel: A1, A2, A3, A4, A5, A6, A7, A8, A9, A10, A11, A12, B1, B2, B3, B4, B5, B6, B7, B8, B9, B10, B11, B12 etc. Please note that this macro was developed using Excel 2003; we expect it to function in newer versions of Excel, but it has not been tested in any version other than 2003.

**Analysis of client protein degradation by western blotting:** Cells were seeded in medium containing 10% FBS for 14-18h and treated with compound for 4-24h, as indicated in the respective figures. For data shown in Figure S3, cells were switched to serum-free medium prior to compound treatment. Cells were lysed in RIPA buffer (BP-115 , Boston Bioproducts, Ashland, MA) containing protease inhibitors (11836153001, Roche Diagnostics, Indianapolis, IN) and phosphatase inhibitors (524624 / 524625, EMD Biochemicals). Protein was quantified using the BCA protein assay (23235, Pierce, Rockford, IL) and 30 ug protein/well (Figure S3) or 15 ug/well (Figure S4) was separated by SDS-PAGE.

Proteins were transferred to nitrocellulose membrane, blocked with Odyssey blocking buffer (927-40000, Li-Cor Biosciences, Lincoln, NE), and were detected with the following antibodies: pERK-T202/Y204 (Cell Signaling Technology 9101), ERK (Cell Signaling Technology 4696), CRAF/RAF-1 (BD Biosciences 610151), BRAF (Santa Cruz Biotechnology sc-166 or sc-9002), GAPDH (Santa Cruz Biotechnology sc-32233), EGFR (Cell Signaling Technology 2646), MET (Cell Signaling Technology 3148), ERBB2 (Cell Signaling Technology 2248), CDK1 (Cell Signaling Technology 9116), CHK1 (Cell Signaling Technology 2360). Primary antibodies were detected and quantified using Li-Cor Odyssey IRdye-conjugated secondary antibodies, a Li-Cor Odyssey scanner and ImageQuant software (GE Healthcare Life Sciences, Piscataway, NJ).

**BrdU Cell Proliferation Assay:** Cells were seeded in 96-well plates in complete growth medium. 14-18h after seeding, cells were treated with compound for 48h and assayed for proliferation using a BrdU (bromo-deoxyuridine, Roche, #10280879001, 20 µM) ELISA assay. After addition of BrdU, cells were incubated for 2-4 h and then fixed with 70% ethanol + 0.1 M NaOH for 30 min. Peroxidase-conjugated anti-BrdU (Roche, #11585860001, 1/2000 in PBS + 1% BSA) was added, and 2-4h later cells were washed 3 times with PBS. Chemiluminescent substrate solution (Pierce, #3707A/B) was added, and the plates were read on the Wallac Victor plate reader. Proliferation IC_50_ values were determined based on a comparison of compound-treatment values to DMSO control values (final DMSO concentration 0.3%).

**Apoptosis – DNA Fragmentation Assay:**  Apoptosis was quantified using a DNA fragmentation assay. Cells were seeded in 96‑well plates in complete growth medium. 14-18h after seeding, cells were treated with compound for 72h: Positive control wells received taxol (paclitaxel), camptothecin, or Adriamycin, while negative control wells received growth medium + 0.3% DMSO media. After the 72h compound treatment, cells assayed for apoptosis using the Cell Death Detection ELISAPLUS kit (Roche, #11920685001). The plates were centrifuged and supernatant discarded. The remaining cell monolayers were lysed, lysates transferred to a streptavidin-coated microtiter plate, and the immunoreagent containing anti-histone and anti-DNA-POD antibodies was added. Following a 2h incubation, the plates were washed with 1× incubation buffer. ATBS substrate solution was then added, and after a 3-5 min incubation, absorbance at 405 nm was measured using the Wallac Victor plate reader (Perkin-Elmer, Waltham, MA). EC_50_ values were calculated based on the absorbance measurement obtained by compound-treated cells versus that of the corresponding positive control wells.
